# Annotations

**Published:** 1895-04-13

**Authors:** 


### Annotations.

### Hereditary Disqualification for Marriage.

A recent breach of promise action brought a question prominently before the public which well deserved more attention from the press than lay journals condescended to give it. A doctor who had been engaged to a young lady, on discovering that her mother had died of phthisis at an early age, refused to complete the contract. The judge and jury took the view that the defence was not put forward in good faith, and gave a verdict against the doctor, with heavy damages. It is probable, however, that if the judge and jury could have been certainly convinced that the doctor refused to marry the young lady on no other ground than that of her presumed hereditary tendency to consumption, though they might still have given the verdict against him, they would have reduced the damages to the lowest amount compatible with justice and common sense. For it is certain that persons of both sexes who have a decided tendency to hereditary consumption ought to refrain from marriage, and this obligation is almost as clearly recognised by the general public as by the medical profession. The obligation is perhaps more especially binding upon women than upon men, at any rate during the period of child-bearing. At that period a double risk is run: a risk to the mother, who often dies of phthisis after giving birth to several children, and leaves them motherless; and a risk to the children so born of themselves becoming consumptives. If it be a duty which only the most urgent circumstances can countervail, for a woman who has a history of hereditary consumption behind her to refrain from marriage, one need not be surprised to find many people maintaining that no persons should be allowed by law to contract marriage in cases where hereditary consumption can be definitely proved to taint both parties to the contract. That is to say, if a man of definitely phthisical history proposes to marry a woman of definitely phthisical history, the law, according to this view, should put its foot down and say "No." A simple statute of this kind, if universally known, would enforce physiological instruction by Act of Parliament; it would check unwise engagements; it would prevent much misery to innocent offspring, and it would considerably limit the actual number of consumptives in a very short period of time.

### Adulterated Milk at Public Institutions.

The last report of the Local Government Board draws attention to a matter which is of considerable importance to public institutions, pointing out that they are apt to be especially victimised by our friend the milkman. Mr. Pattinson, the public analyst for Newcastle-on-Tyne, after an examination of the milk supplied to the various public institutions of the city, writes: "It was found that some of these institutions were supplied with genuine milk, but others were being supplied with grossly adulterated milk. The milk supplied to the workhouse contained 18 per cent. of added water; that supplied to the barracks contained 36 per cent. of added water; two samples of the milk supplied to the Girls' Orphanage, Town Moor, contained respectively 22 and 18 per cent. of added water; two samples of the milk supplied to the Deaf and Dumb Asylum, Town Moor, contained respectively 18 and 14 per cent. of added water; and three samples taken from the milk supply of the Fleming Memorial Children's Hospital were each adulterated with 30 per cent. of added water." These instances prove the necessity for a strict watch being kept by the governing bodies of all such institutions, in order to insure the purity of the milk supplied to the inmates under their charge. In regard to the exercise of such supervision it is perhaps as well to point out, as is done in the report referred to, that adulteration of milk is in many places carried on to a much larger extent on Sundays than on other days of the week. One analyst, writing on the subject, says, "It is evident that on ordinary days one may expect to find one out of every six milks that are purchased to be adulterated, while on Sundays the probability is that about one out of every three milks has been tampered with. Sunday with the poorer classes is a great day for preparing milk puddings." Sunday then is the day when matrons, cooks, and stewards should be especially on the watch. If there is the slightest cause for suspicion it is a simple matter to fill a half-pint bottle out of the bulk supplied, for examination by the dispenser or by the public analyst, and if the sample is taken in the presence of the milkman it will generally have the effect of making him careful in the future. The complete analysis of milk is a very delicate process, but milkmen know well enough that rough tests, which would quite justify a manager in refusing to renew a contract, are very easily applied.

### A Patent for Antitoxin.

A controversy of considerable importance to the future of medical practice in civilised countries is now being carried on in the pages of a contemporary. Dr. Aronson claims, or his agent claims for him, that he produced antitoxin "for the first time from the horse," and "intensified the virulence of the poison by processes which he described in the *Berlin Klinische Wochenschrift*." For antitoxin thus produced Dr. Aronson has been advised by his agent in this country to apply for a patent, and thus to turn "Aronson's antitoxin" into a patent medicine, by means of the English Patent Office. There will not be two opinions among British practitioners as to the entire undesirability of all "patent medicines" of every class. A "patent medicine" is, in fact, a ridiculous absurdity and a fraud. But that a legally qualified practitioner should attempt to take out, or should succeed in taking out, a patent for a "remedy" is a thing which, we venture to think, is unheard of among British practitioners. The practice of all right-minded medical men in this country is to make full disclosure of every method of treatment which has proved serviceable, not by patenting it but by publishing it for the benefit of all. This is as manifestly in the interests of the health of the whole community as in those of the honesty, candour, and disinterestedness of the medical profession. This "honest, candid, and disinterested" principle of medical practice is, we repeat, as essential to the honour of the profession as it is to the well-being of the public; and it is a principle which must be conserved at all risks. It is a principle which the profession would not allow a British practitioner to violate on any consideration; and it is a principle which a foreigner, likewise, must not be allowed to violate. The medical profession is the natural guardian of medical ethics in this country; and if need be, it must rise to the last man in order to impress its judgment and will upon Parliament in a question of such first-rate and wide-reaching public and professional importance.

